# A Pyrone Glucoside from *Maerua angolensis* Induces Caspase-Dependent Apoptosis and Targets AKT1, PARP-1, and Caspase-7 in Triple-Negative Breast Cancer

**DOI:** 10.3390/biom16060861

**Published:** 2026-06-11

**Authors:** Jamila Aminu, Amina Jega Yusuf, Bor-Jang Hwang, Sonia Kamran, Nasiru Abdullahi, Adamu Jibril Alhassan, John Obadipe, Valerie Odero-Marah, Hajjagana Hamza, Abdullahi Ibrahim Uba, James Wachira, Jiangnan Peng

**Affiliations:** 1 Department of Chemistry, Morgan State University, 1700 E. Cold Spring Lane, Baltimore, MD 21251, USA; jamila.aminu@morgan.edu (J.A.); amina.yusuf@morgan.edu (A.J.Y.); sokam1@morgan.edu (S.K.); 2 Department of Biochemistry, Bayero University, P.M.B. 3011, Gwarzo Road, Kano, Nigeria; nabdullahi.bch@buk.edu.ng (N.A.); ajalhassan.bch@buk.edu.ng (A.J.A.); 3 Department of Biochemistry, Gombe State University, P.M.B. 127, Tudun Wada, Gombe, Gombe State, Nigeria; hghamzah@gsu.edu.ng; 4 Department of Biology, Morgan State University, 1700 E. Cold Spring Lane, Baltimore, MD 21251, USA; bor-jang.hwang@morgan.edu (B.-J.H.); jooba1@morgan.edu (J.O.); valerie.odero-marah@morgan.edu (V.O.-M.); 5 Department of Biostatistics and Medical Informatics, Faculty of Medicine, İstinye University, 34396 Istanbul, Türkiye; abdullahi.uba@istinye.edu.tr

**Keywords:** *Maerua angolensis*, cytotoxic activity, pyrone glucoside, MDA-MB-468 cells, triple-negative breast cancer

## Abstract

Triple-negative breast cancer (TNBC) is an aggressive subtype lacking effective targeted therapies, highlighting the need for new anticancer agents. Natural products remain a valuable source of bioactive compounds with diverse mechanisms of action. In this study, a pyrone glucoside, 7-hydroxymaltol-3-*O*-*β*-D-glucoside, was isolated from the methanolic leaf extract of *Maerua angolensis* and evaluated for its anticancer activity against TNBC cells. Structural elucidation was achieved using NMR and LC–MS analyses. Both the crude extract and the isolated compound exhibited dose-dependent cytotoxicity against MDA-MB-468 cells, with IC_50_ values of 2.94 and 0.78 µg/mL, respectively, while showing reduced toxicity toward MCF10A normal cells. Mechanistic studies revealed induction of apoptosis, evidenced by activation of caspase-9 and caspase-7 and PARP cleavage. Confocal imaging further demonstrated lysosomal disruption and nuclear morphological alterations consistent with stress-associated cell death. Gene expression analysis indicated minimal involvement of the PI3K/AKT/mTOR pathway. Molecular docking showed favorable binding of the compound to AKT1, PARP-1, and caspase-7, suggesting a multi-target mode of action. ADMET analysis indicated low oral bioavailability but a favorable safety profile. These findings highlight the potential of this compound as a lead for TNBC therapy.

## 1. Introduction

Breast cancer (BC) is a leading cause of cancer-related mortality worldwide, and in the United States accounts for over one-third of new cancer diagnoses in women [1]. BC is classified based on the expression of estrogen receptor (ER), progesterone receptor (PR), and human epidermal growth factor receptor 2 (HER2/ERBB2), which guides therapeutic decision-making [2]. Current treatments include surgery, radiation, chemotherapy, and targeted therapies such as hormone therapy, kinase inhibitors, and biologics [3]. Tumors expressing HER2 or hormone receptors may be treated with targeted agents, whereas the absence of validated molecular targets in triple-negative breast cancer (TNBC) necessitates reliance on chemotherapy [4]. Although specific genetic alterations, including *BRCA1/BRCA2* mutations and dysregulation of the PI3K/AKT/mTOR pathway, have enabled targeted therapeutic strategies in subsets of patients, resistance and disease relapse remain common [5,6,7,8,9,10,11].

TNBC accounts for approximately 10–15% of BC cases and is characterized by aggressive growth, high metastatic potential, and pronounced molecular heterogeneity, complicating targeted therapy development [4,12,13]. Recent advances include antibody–drug conjugates, immune checkpoint inhibitors for PD-L1–positive tumors, PARP inhibitors for *BRCA*-mutant tumors, and inhibitors of the PI3K/AKT/mTOR signaling pathway [3,4,14,15,16,17]. Gene expression profiling has further enabled the sub-classification of TNBC into molecular subtypes, providing a framework for pathway-directed therapeutic strategies [18]. Despite these advances, treatment responses are often incomplete, resistance develops frequently, and metastatic TNBC remains associated with poor clinical outcomes [19].

Natural products and their derivatives continue to contribute substantially to small-molecule drug discovery, with many FDA-approved anticancer agents originating from or inspired by natural product scaffolds [20]. Many clinically important anticancer drugs originate from plants, bacteria, and fungi, underscoring the value of bioactive secondary metabolites in drug discovery [21,22]. Screening of chemically diverse natural product libraries and ethnopharmacological knowledge has facilitated the identification of compounds with anticancer potential [23,24,25]. Herbal medicines are rich in flavonoids, alkaloids, terpenoids, phenolics, and related metabolites that can modulate oncogenic signaling pathways and continue to attract interest for anticancer drug development [7,26,27,28].

*Maerua angolensis* DC (Capparaceae) is native to tropical Africa and is widely used in traditional medicine for the treatment of various ailments, including inflammatory conditions and cancer [29]. Phytochemical studies indicate that *M. angolensis* contains diverse secondary metabolites, including flavonoids, alkaloids, tannins, terpenoids, phenolics, and anthraquinones [30,31,32]. Extracts of the plant have demonstrated antioxidant, anticonvulsant, and anti-inflammatory activities in experimental models [33,34,35]. Importantly, several compound classes reported in *M. angolensis* are known to regulate cancer-associated pathways, including PI3K/AKT/mTOR and Wnt/β-catenin signaling [36]. However, the anticancer potential of this species remains underexplored at the compound level. Accordingly, we report the isolation and structural elucidation of a pyrone glucoside, 7-hydroxymaltol-3-*O*-*β*-D-glucoside, from the leaves of *M. angolensis*. We evaluate its cytotoxic activity against TNBC cells and investigate its mechanism of action through biochemical, imaging, and gene expression analyses. Furthermore, molecular docking studies were performed to explore its interactions with key therapeutic targets, including AKT1, PARP-1, and caspase-7, and to provide insights into its potential multi-target anticancer activity.

## 2. Materials and Methods

### 2.1. General Experimental Procedures

All solvents used for extraction, chromatographic, and spectroscopic analysis were of analytical or HPLC grade from Fisher Scientific (Waltham, MA, USA). Methanol, dichloromethane, ethyl acetate, hexane, and other solvents were purchased from commercial suppliers and used without further purification. Flash chromatography was performed using a Biotage Isolera One system equipped with UV detection (Biotage AB, Charlotte, NC, USA). TLC was carried out on silica gel 60 F_254_ plates, and spots were visualized under UV light at 254 and 365 nm, followed by staining with 10% sulfuric acid in methanol and heating. 1D- and 2D-NMR spectra were recorded in CD_3_OD on a 400 MHz Varian MR-400 NMR spectrometer (Varian, Inc. Palo Alto, CA, USA), and chemical shifts were referenced to the residual solvent signals at δ_H_ 3.31 ppm and δ_C_ 49.0 ppm. Chemical shifts (δ) are reported in parts per million (ppm) relative to residual solvent signals, and coupling constants *(J*) are reported in hertz (Hz). HR–MS analysis was performed on Agilent QToF 6545 instruments (Agilent Technologies, Inc., Santa Clara, CA, USA), equipped with an electrospray ionization (ESI) source operated in positive-ion mode. Analytical HPLC analysis was carried out on a Shimadzu LC-2050C 3D liquid chromatography system (Shimadzu Corporation, Kyoto, Japan) using a reversed-phase C18 column (150 × 4.6 mm, 5 µm particle size) to assess compound purity. Detection was achieved using a UV detector at 215 nm and 254 nm (Shimadzu LC-2050C, Kyoto, Japan). The mobile phase consisted of water (A) and methanol (B). A gradient elution program was applied as follows: 10% B from 0.0 to 8.0 min, a linear increase to 100% B from 8.0 to 12.0 min, followed by re-equilibration at 10% B until 15.0 min. The flow rate was set to 0.8 mL/min. Confocal fluorescence imaging was performed using a Leica Stellaris 5 confocal microscope (Leica, Wetzlar, Germany). Fluorescence quantification and image analysis were conducted using Fiji (ImageJ 1.54f). Immunoblot detection was carried out using enhanced chemiluminescence reagents, and signals were visualized using a digital imaging system. Quantitative real-time PCR was performed using a Bio-Rad CFX96 real-time PCR detection system.

### 2.2. Plant Material

Leaves of *Maerua angolensis* were collected from Deba village, Yamaltu/Deba Local Government Area, Gombe State, Nigeria, in the month of November 2024. The plant material was authenticated at the Herbarium Unit, Department of Plant Science, Faculty of Science, Gombe State University, Gombe, Nigeria, where a voucher specimen was deposited (GSUH/240). The leaves were air-dried, pulverized, and stored until extraction.

### 2.3. Extraction and Isolation

Air-dried and pulverized leaves of *M. angolensis* were extracted by maceration with methanol (10:1, *v*/*w*) at room temperature for 24 h. The extract was filtered, and the plant residue was re-extracted twice under identical conditions. Combined filtrates were concentrated under reduced pressure at ≤40 °C to afford the crude methanolic leaf extract (MALM). A portion of MALM (4.0 g) was subjected to flash column chromatography using a Biotage Isolera One system fitted with a SNAP KP-Sil silica cartridge (120 g) (Charlotte, NC, USA). The sample was pre-adsorbed onto Celite^®^, dried, and loaded onto the column. Elution was performed using a stepwise gradient of dichloromethane–methanol (100% DCM 5CV, 95:5 DCM:MeOH 3CV, 90:10 DCM:MeOH 2CV, 80:20 DCM:MeOH 3CV, 70:30 DCM:MeOH 2CV, 50:50 DCM:MeOH 2CV, 100% MeOH 2CV), at a flow rate of 50 mL/min. Fractions were monitored by TLC on silica gel plates and combined based on similar chromatographic profiles to yield 26 pooled fractions (MALM-F1–MALM-F26). Fractions MALM-F19 and MALM-F20 were further purified by crystallization from methanol. The resulting crystals were collected to afford FC20C as white crystals.

### 2.4. Spectral Data

*7-hydroxymaltol-3-O-β-glucoside* (FC20C). White crystalline solid, m.p. 147.3~149.7. [α]_D_ −40º (c = 0.1). ^1^H-NMR (400 MHz, CD_3_OD): δ_H_ 6.50 (d, *J* = 5.6 Hz, H-5), 8.10 (d, *J* = 5.6, H-6), 4.75 (d, *J* = 13.6, H-7), 4.61 (d, *J* = 13.6, H-7), 4.79 (d, *J* = 7.2 Hz, H-1′), 3.28 (H-2), 3.38 (H-3′), 3.29 (H-4′), 3.41 (H-5′), 3.86 (dd, *J* = 11.8, 2.0 Hz, H-6′), 3.68 (dd, *J* = 11.8, 5.2 Hz, H-6′). ^13^C-NMR (100 MHz, CD_3_OD): δ_C_ 162.3 (C-2), 141.3 (C-3), 176.2 (C-4), 116.3 (C-5), 156.2 (C-6), 56.3 (C-7), 103.2 (C-1′), 76.9 (C-2′), 73.8 (C-3′), 69.9 (C-4′), 76.4 (C-5′), 61.2 (C-6′). HR–MS (ESI^+^) *m*/*z:* 305.0865 [M + H]^+^, Calculated for C_12_H_17_O_9_ (305.0873). Analytical HPLC analysis indicated >95% purity.

### 2.5. Cell Culture and Chemical Reagents

Human triple-negative breast cancer cells (MDA-MB-468) and non-tumorigenic breast epithelial cells (MCF10A) were obtained from the Sidney Kimmel Comprehensive Cancer Center, Johns Hopkins University. Cells were maintained at 37 °C in a humidified incubator with 5% CO_2_. MDA-MB-468 cells were cultured in Dulbecco’s Modified Eagle Medium (DMEM) supplemented with 10% fetal bovine serum and penicillin–streptomycin (100 IU/mL and 100 μg/mL, respectively). MCF10A cells were cultured in DMEM/Ham’s F-12 medium supplemented with 5% horse serum, insulin (10 μg/mL), epidermal growth factor (20 ng/mL), hydrocortisone (0.5 μg/mL), cholera toxin (100 ng/mL), and penicillin–streptomycin (100 IU/mL and 100 μg/mL). LysoTracker dyes, DRAQ5, and DRAQ7 were purchased from Thermo Fisher Scientific. Antibodies used for immunoblot analysis were obtained from Cell Signaling Technology. All other reagents were obtained from commercial suppliers and used without further purification.

### 2.6. Cytotoxicity Assay

Cell viability was assessed using a colorimetric assay following 48 h of treatment. MDA-MB-468 and MCF10A cells (3000 per well) were seeded into 96-well plates and allowed to attach overnight. Cells were treated with MALM or purified 7-hydroxymaltol-3-*O*-*β*-D-glucoside (FC20C) at concentrations ranging from 0 (DMSO vehicle), 0.78 to 100 μg/mL, and 5 ng/mL paclitaxel. Stock solutions were prepared in DMSO and diluted in culture medium such that the final DMSO concentration did not exceed 0.1% (*v*/*v*). After 48 h of incubation, cell viability was determined by crystal violet staining. Media were aspirated, 100 μL of 0.5% crystal violet (in 20% methanol) was added to the wells, and the wells were incubated at room temperature with gentle agitation for 30 min. Plates were washed gently in tap water (submerged in a water container, avoiding direct cell flush) until the background was completely removed. Plates were air-dried on the bench overnight. Trapped crystal violet within the surviving cells was extracted with 10% glacial acetic acid and gently shaken for 20 min at room temperature, and the absorbance was measured at 590 nm using a BioTek plate reader. Cell viability was expressed as a percentage relative to DMSO-treated control cells. Each treatment was performed in sextuplicate, and experiments were repeated independently at least twice [37]. Concentration–response curves were generated, and the IC50 values were computed using the online AAT Biorequest IC50 Calculator, accessible at https://www.aatbio.com/tools/ic50-calculator (accessed on 9 October 2025). Statistical significance was evaluated using one-way analysis of variance (ANOVA).

### 2.7. Confocal Microscopy

MDA-MB-468 cells were seeded in 8-well glass-bottom chamber slides and allowed to attach for 24 h prior to treatment. Cells were treated with MALM or purified 7-hydroxymaltol-3-*O*-*β*-D-glucoside (FC20C) at their respective IC_50_ concentrations. Following treatment, lysosomal compartments were stained using LysoTracker (Thermo Fisher Scientific) according to the manufacturer’s instructions. Nuclear staining was performed using DRAQ5 or DRAQ7, where indicated. Fluorescence images were acquired using a Leica Stellaris 5 confocal microscope (Wetzlar, Germany). Quantitative fluorescence analysis was performed using Fiji (ImageJ 1.54f). Organelle segmentation was carried out using the StarDist plugin, and the processed data were analyzed using GraphPad Prism(10.1).

### 2.8. Immunoblot Analysis

MDA-MB-468 cells were treated with MALM or purified 7-hydroxymaltol-3-*O*-β-D-glucoside (FC20C) at the indicated concentrations. Following treatment, cells were washed with phosphate-buffered saline and lysed in ice-cold lysis buffer containing protease inhibitors. Cell lysates were clarified by centrifugation, and protein concentrations were determined using a bicinchoninic acid (BCA) assay. Equal amounts of protein (30 μg) were resolved by SDS–PAGE using 4–20% Tris–glycine gels and transferred onto polyvinylidene difluoride (PVDF) membranes. Membranes were blocked with 5% nonfat milk in Tris-buffered saline containing 0.1% Tween 20 and incubated overnight at 4 °C with primary antibodies against caspase-3, cleaved caspase-3, caspase-7, cleaved caspase-7, caspase-9, cleaved caspase-9, PARP-1, cleaved PARP-1, or β-actin. After washing, membranes were incubated with horseradish peroxidase-conjugated secondary antibodies. Immunoreactive bands were visualized using enhanced chemiluminescence reagents and detected using a digital imaging system.

### 2.9. Quantitative Real-Time PCR (qRT-PCR)

Total RNA was isolated from treated and control MDA-MB-468 cells using the RNeasy Mini Kit according to the manufacturer’s instructions. RNA concentration and integrity were assessed spectrophotometrically, and only samples of high quality were used for subsequent analysis. Complementary DNA (cDNA) was synthesized from total RNA using a reverse transcription kit following the supplier’s protocol. Quantitative real-time PCR was performed using SYBR Green chemistry on a Bio-Rad CFX96 real-time PCR detection system. Reactions were carried out in a total volume of 20 μL containing cDNA template and gene-specific primers. Amplification conditions consisted of an initial denaturation step followed by 40 cycles of denaturation and annealing/extension. Melt-curve analysis was performed to verify amplification specificity. Gene expression levels were normalized to β-actin as the internal reference gene. Relative expression was calculated using the 2^ΔΔCq^ method. Primer sequences used in this study are provided in Supplementary Table S1. All experiments were performed in triplicate, and statistical analysis was conducted using two-way analysis of variance (ANOVA) with appropriate post hoc testing.

### 2.10. Molecular Docking

The molecular docking studies were conducted to evaluate the binding potential of a selected plant-derived ligand against AKT1 (PDB ID: 3OCB) [38], caspase-7 (PDB ID: 8DJ3) [39], and PARP-1 (PDB ID: 7KK4) [40]. The three-dimensional crystal structures of the target proteins were retrieved from the Protein Data Bank (PDB). Protein structures were prepared using the Protein Preparation Wizard in Schrödinger’s Maestro suite (2019.2). This preparation involved the addition of missing hydrogen atoms, assignment of correct bond orders, removal of crystallographic water molecules beyond 5 Å from the active site, and optimization of hydrogen bonding networks. The protein protonation states were adjusted for pH 7.4, and restrained energy minimization was performed using the OPLS3 force field to relieve steric clashes [41].

The structure of 7-hydroxymaltol-3-*O*-β-D-glucoside was retrieved from the Pubchem database and energy-minimized using LigPrep, generating relevant tautomeric and ionization states at physiological pH. A receptor grid for each protein was generated centered on the active site residues identified from the literature and co-crystallized ligands. Glide docking was performed using extra precision (XP) docking [42]. Docking results were evaluated based on Glide score, hydrogen bonding interactions, hydrophobic contacts, π–π stacking, and other non-covalent interactions. Visualization and analysis of ligand–protein interactions were performed using Maestro Viewer, highlighting key residues contributing to binding affinity.

### 2.11. In Silico ADMET Prediction

The pharmacokinetic and drug-likeness properties of 7-hydroxymaltol-3-*O*-β-D-glucoside were evaluated using the SwissADME online platform. The canonical SMILES of the compound was retrieved from chemical databases and used as input for the analysis. SwissADME provides a comprehensive assessment of absorption, distribution, metabolism, and excretion (ADME) parameters, along with physicochemical descriptors [43]. Key parameters examined included molecular weight, lipophilicity (LogP), water solubility, hydrogen bond donors and acceptors, topological polar surface area (TPSA), and flexibility (rotatable bonds). Pharmacokinetic predictions such as gastrointestinal (GI) absorption, blood–brain barrier (BBB) permeability, P-glycoprotein (P-gp) substrate specificity, and cytochrome P450 (CYP) enzyme inhibition profiles were also analyzed. Additionally, drug-likeness was evaluated based on established rules, including Lipinski.

### 2.12. Statistical Analysis

All data are presented as mean ± standard deviation (SD). Statistical analyses were performed using GraphPad Prism (10.1). Comparisons among multiple groups were conducted using one-way or two-way analysis of variance (ANOVA) with appropriate post hoc tests. A *p* value < 0.05 was considered statistically significant.

## 3. Results and Discussion

### 3.1. Isolation and Structural Elucidation of FC20C

Fractionation of the MALM led to the crystallization of a major constituent, designated FC20C. The compound was obtained as a white crystalline solid, and its purity was confirmed by analytical HPLC, which showed a single symmetrical peak (Supplementary Figure S1). HRMS (ESI^+^) analysis of FC20C showed a protonated molecular ion at *m*/*z* 305.0865 [M + H]^+^, consistent with the molecular formula C_12_H_16_O_9_ calculated for 305.0873, supporting its identification as 7-hydroxymaltol-3-*O*-*β*-D-glucoside (Supplementary Figure S2). The structure of FC20C was elucidated using comprehensive 1D and 2D-NMR spectroscopic analyses, including ^1^H NMR, ^13^C NMR, DEPT, COSY, and HMBC experiments (Table 1). The ^1^H-NMR spectrum of FC20C (CD_3_OD, 400 MHz) revealed distinctive resonances attributable to both the aglycone and a sugar moiety [44,45]. Two well-defined aromatic doublets were observed at *δ_H_* 6.50 (*d*, *J* = 5.6 Hz, H-5) and *δ_H_* 8.10 (*d*, *J* = 5.6 Hz, H-6), showing typical ortho-coupling, which is indicative of adjacent protons on an aromatic ring. These resonances suggest a 1,2-disubstituted aromatic system, frequently seen in phenolic or cinnamic-type structures [45]. Additionally, a pair of diastereotopic methylene doublets appeared at *δ_H_* 4.75 and 4.61 (*d, J* = 13.6 Hz, H-7), consistent with a methylene group attached to an sp^2^-hybridized carbon adjacent to the aromatic moiety [44]. In the sugar region, the anomeric proton signal observed at *δ_H_* 4.79 (d, *J* = 7.2 Hz, H-1′) exhibited a relatively large coupling constant, characteristic of a trans-diaxial relationship between H-1′ and H-2′ in glucopyranosides, thereby supporting a β-glycosidic configuration. A series of signals at *δ_H_* 3.28–3.86 corresponded to sugar protons H-2′ to H-6′. The two double doublets at *δ_H_* 3.86 (*J* = 11.8, 2.0 Hz) and 3.68 (*J* = 11.8, 5.2 Hz) confirmed the presence of a terminal CH_2_OH group (H-6′). Thus, the chemical shift values strongly support the presence of an aromatic aglycone conjugated to a hexose sugar through a β-glycosidic bond [46].

The ^13^C NMR spectrum of FC20C exhibited 12 distinct carbon resonances. The aglycone region indicated three downfield signals at *δ_C_* 176.2 (C-4), 162.7 (C-2), and 141.3 (C-3), indicative of a conjugated carbonyl (C-4) and two sp^2^ carbons (C-2 and C-3) likely involved in π-conjugation [44,47]. Other aromatic carbons detected at *δ_C_* 156.2 and 116.3 were assigned to (C-6) and (C-5), respectively. The signal at *δ_C_* 56.3 corresponded to hydroxymethyl(C-7), consistent with the diastereotopic proton signals observed in the ^1^H-NMR spectrum. For the sugar unit, the anomeric carbon at *δ_C_* 103.3 (C-1′) further confirmed the β-linkage, and other sugar carbons resonated between *δ_C_* 61.2 and 76.9 (C-2′—C-6′), typical of a glucose moiety [44,45]. The downfield anomeric carbon signal also aligned with *O*-glycosidic substitution on the aglycone. DEPT experiments confirmed the nature and multiplicities of the different carbons observed in FC20C; signals at *δ_C_* 116.3 (C-5) and 156.2 (C-6) were identified as CH groups, while the resonance at *δ_C_* 56.3 (C-7) corresponded to a methylene (CH_2_) group. In contrast, the signals at *δ_C_* 176.2 (C-4), 162.7 (C-2), and 141.3 (C-3) appeared as quaternary carbons, consistent with non-protonated, electron-rich centers of the aromatic aglycone [44,47]. Within the sugar region, CH signals were clearly assigned to C-1′ through C-5′, whereas C-6′ was characterized as a methylene carbon at *δ_C_* 61.2, confirming the presence of a terminal CH_2_OH group typical of a hexopyranosyl unit [48].

**Figure 1:**
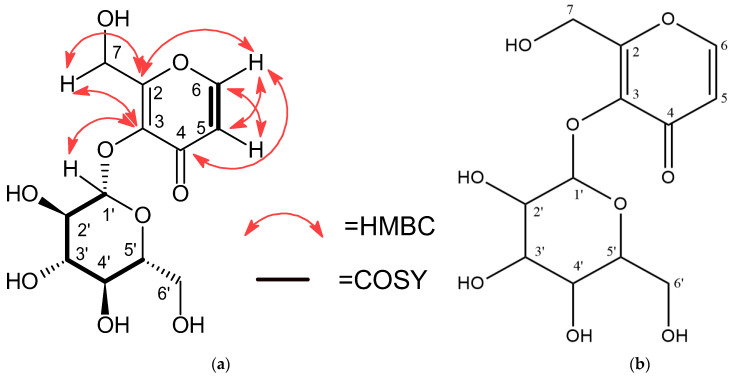
(**a**) Key ^1^H–^1^H COSY (red) and HMBC (blue) correlations observed for FC20C. (**b**) Chemical structure of FC20C (7-hydroxymaltol-3-*O*-*β*-D-glucoside).

The COSY spectrum showed a clear cross-peak between H-5 (*δ_H_* 6.50) and H-6 (*δ_H_* 8.10), which confirmed the ortho-coupled relationship on the aromatic ring. This is consistent with the substitution pattern of a 1,2-disubstituted benzene ring [44,45]. Strong sequential correlations were observed from H-1′ to H-2′, and from H-2′ through to H-6′ within the sugar region, further confirming the connectivity of the hexose ring, typical of *β*-D-glucopyranosyl structure [44]. HMBC correlations were crucial for defining the aglycone–sugar linkage in FC20C. H-5 showed long-range correlations with C-3, C-4, and C-6, while H-6 correlated with C-2, C-4, and C-5, which established their relative positions on the aromatic ring. The correct assignment of H-7 on the aromatic moiety was confirmed via its correlations to C-2 and C-3. Importantly, H-1′ (anomeric proton) exhibited a clear long-range correlation to C-3 of the aglycone, establishing the point of attachment of the sugar unit through an *O*-glycosidic bond, typical of hydroxylmaltol (Figure 1a) [44,48]. Based on the 1D- and 2D-NMR data of FC20C, and comparison with related data in the literature [44,48,49], the structure of the compound was confirmed to be 7-hydroxymaltol-3-*O*-*β*-D-glucoside (Figure 1b).

### 3.2. Cytotoxic Activity of MALM and FC20C

**Figure 2:**
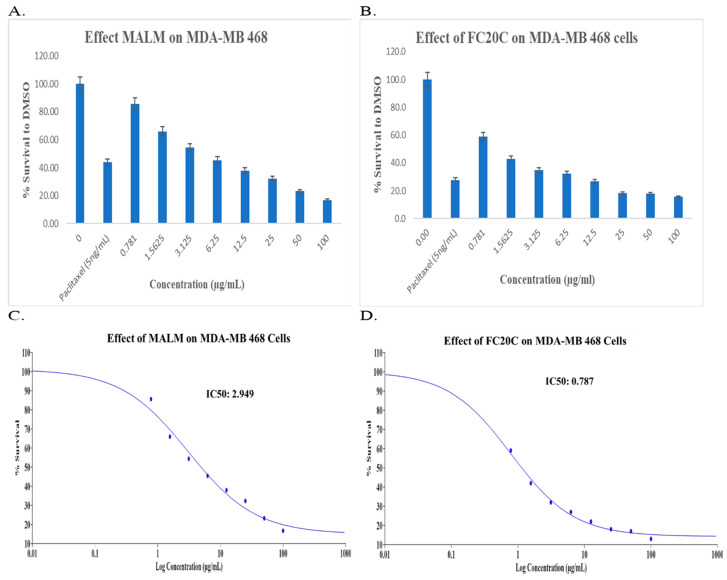
Dose-dependent cytotoxic effects of MALM and FC20C on MDA-MB-468 cells. Cells were treated for 48 h with increasing concentrations of MALM or FC20C and 5 ng/mL Paclitaxel, and viability was assessed by crystal violet assay. Data are expressed as mean ± SD (n = 3). IC_50_ values were determined by nonlinear regression. (**A**) Cells treated with MALM. (**B**) Cells treated with FC20C. (**C**) Dose responding curve for MALM. (**D**) Dose responding curve for FC20C.

**Figure 3:**
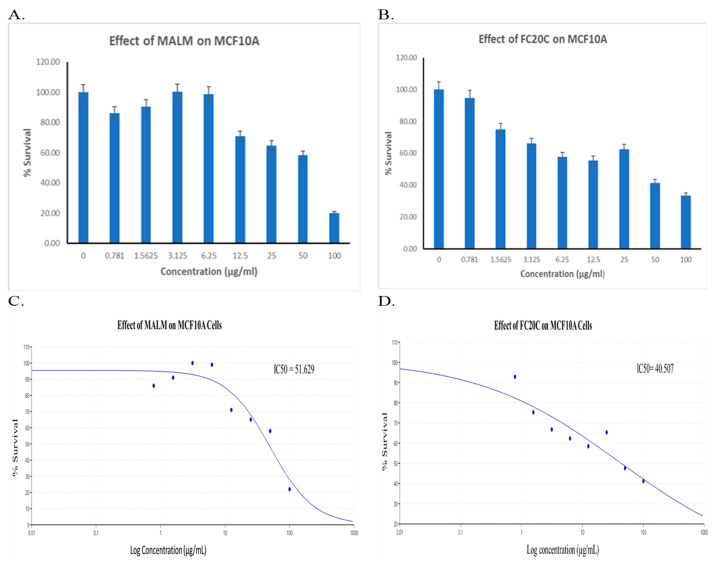
Reduced cytotoxicity of MALM and FC20C in MCF10A normal breast epithelial cells. Cells were treated for 48 h with MALM or FC20C, and viability was assessed by crystal violet assay. Data are expressed as mean ± SD (n = 3). (**A**) Cells treated with MALM. (**B**) Cells treated with FC20. (**C**) Dose responding curve for MALM. (**D**) Dose responding curve for FC20C.

The cytotoxic effects of MALM and purified FC20C were evaluated against MDA-MB-468 triple-negative breast cancer (TNBC) cells using a crystal violet assay following 48 h exposure. Both samples exhibited dose-dependent inhibition of cell viability (Figure 2). MALM displayed an IC_50_ value of 2.94 µg/mL, whereas FC20C showed enhanced potency with an IC_50_ value of 0.78 µg/mL, corresponding to approximately 2.5 µM. To assess selectivity, cytotoxicity was also evaluated in MCF10A normal breast epithelial cells. At concentrations effective against MDA-MB-468 cells, both MALM and FC20C showed reduced effects on MCF10A cells with the IC_50_ values of 51.62 µg/mL and 40.50 µg/mL (Figure 3), indicating preferential activity toward TNBC cells. Pyrone derivatives, including glycosylated analogs, have been reported from several plant species and are increasingly recognized for their diverse biological activities. For example, pyrone glucosides isolated from *Helichrysum italicum* and *Pachira glabra* have demonstrated bioactivity in pharmacological assays, supporting this structural class as a source of biologically relevant metabolites [46,50,51]. In this context, the isolation of 7-hydroxymaltol-3-*O*-*β*-D-glucoside from *M. angolensis* expands the chemical space of plant-derived pyrone glycosides. Although *M. angolensis* is widely used in African traditional medicine to treat diverse ailments, including infectious diseases, inflammatory conditions, and cancer [29], its anticancer potential has not previously been substantiated at the compound level. In the present study, both MALM and the isolated compound FC20C exhibited measurable cytotoxicity against TNBC cells, with IC_50_ values within a range commonly reported for natural product leads in early-stage screening assays. While IC_50_ values alone do not necessarily predict in vivo efficacy, they provide an initial indication of bioactivity and justify further mechanistic evaluation [52].

### 3.3. Induction of Apoptosis via Caspase Activation

**Figure 4:**
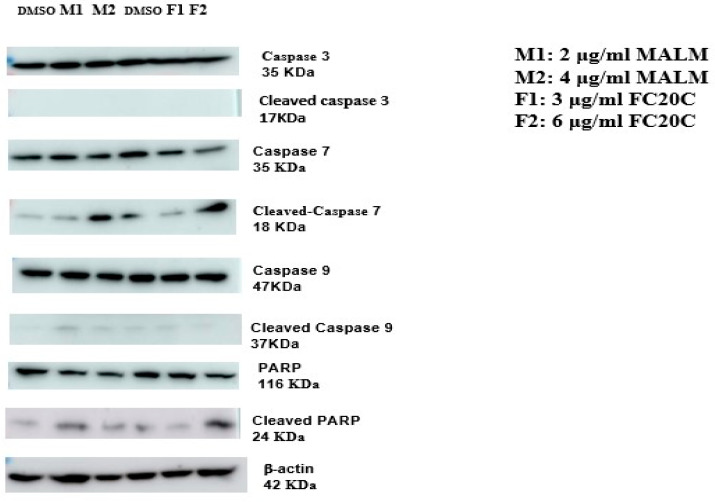
MALM and FC20C induce caspase activation in MDA-MB-468 cells. Cells were treated with MALM (M1, 2 µg/mL; M2, 4 µg/mL) or purified 7-hydroxymaltol-3-*O*-*β*-D-glucoside (FC20C; F1, 3 µg/mL; F2, 6 µg/mL), and total cellular extracts were analyzed by immunoblotting. Increased levels of cleaved caspase-7 (18 kDa), cleaved caspase-9 (37 kDa), and cleaved PARP fragments (24 kDa) were observed in treated cells compared to DMSO controls. β-Actin (42 kDa) was used as a loading control. Original Western blot images can be found in Figure S1.

To investigate whether the observed cytotoxicity was associated with apoptotic cell death, activation of caspases and cleavage of poly(ADP-ribose) polymerase-1 (PARP-1) were examined by immunoblot analysis. Caspases are central regulators of programmed cell death and are broadly classified into initiator caspases, such as caspase-9 and caspase-8, and effector caspases, including caspase-3, -6, and -7 [53]. Activation of caspase-9 is a hallmark of the mitochondrial-mediated intrinsic apoptotic pathway, initiated by cytochrome *c* release and apoptosome assembly [54]. Treatment of MDA-MB-468 cells with MALM or purified 7-hydroxymaltol-3-*O*-*β*-D-glucoside (FC20C) resulted in increased levels of cleaved caspase-9 and cleaved caspase-7, particularly at higher concentrations (Figure 4), indicating engagement of the intrinsic apoptotic pathway. In parallel, accumulation of the characteristic 24 kDa PARP-1 cleavage fragments was observed in treated cells, further supporting apoptosis induction. In contrast, cleaved caspase-3 was minimally detected under the experimental conditions, suggesting that caspase-7 functions as the predominant executioner caspase in this cellular context.

Caspase-7 has been reported to act as a potent amplifier of apoptotic signaling and to cleave PARP-1 more efficiently than caspase-3 in certain cancer cell types [55,56]. Consistent with these reports, the biochemical profile observed here indicates preferential reliance on caspase-7-mediated execution of apoptosis in MDA-MB-468 cells. The activation of caspase-9 together with downstream PARP-1 cleavage supports a mitochondria-mediated apoptotic mechanism contributing to the cytotoxic effects of MALM and FC20C in TNBC cells.

Although MDA-MB-468 cells can undergo classical apoptosis [57,58], it is noteworthy that some anticancer agents induce cell death through apoptosis-independent mechanisms [59,60,61]. In the present study, the concomitant lysosomal and nuclear alterations observed by confocal microscopy suggest that MALM and FC20C may engage multiple stress-associated cell death pathways in addition to intrinsic apoptosis. Further studies will be required to define the upstream molecular events and to delineate the relative contribution of alternative cell death mechanisms.

### 3.4. Effects of MALM and FC20C on Lysosomal Function and Cell Morphology

**Figure 5:**
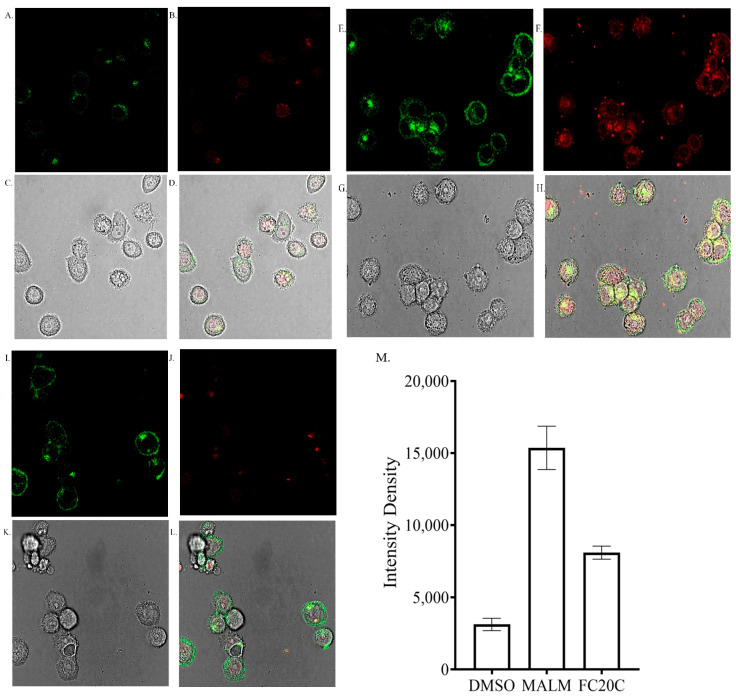
MALM and FC20C disrupt lysosomal integrity in MDA-MB-468 cells. Cells were cultured in chamber slides and treated overnight with MALM or purified 7-hydroxymaltol-3-*O*-*β*-D-glucoside (FC20C) at their respective IC50 concentrations. DMSO-treated cells are shown in panels (**A**–**D**), MALM-treated cells in panels (**E**–**H**), and FC20C-treated cells in panels (**I**–**L**); panel (**M**) shows MALM-treated cells at higher intensity. Lysosomal compartments were visualized using LysoTracker, and nuclei were counterstained with DRAQ5. Images were acquired by confocal microscopy at 63× magnification.

Lysosomal alterations induced by MALM and purified 7-hydroxymaltol-3-*O*-*β*-D-glucoside (FC20C) were evaluated using LysoTracker staining and confocal microscopy (Figure 5). Compared to DMSO-treated controls (Figure 5A–D), cells treated with MALM (Figure 5E–H) or FC20C (Figure 5I–L) exhibited a marked increase in LysoTracker fluorescence intensity, indicating accumulation of acidic vesicular compartments. Quantitative analysis confirmed significantly elevated fluorescence intensity in treated cells relative to controls (Figure 5M). These observations suggest disruption of lysosomal homeostasis following exposure to MALM and FC20C, a phenomenon commonly associated with cellular stress induced by cytotoxic agents [62].

**Figure 6:**
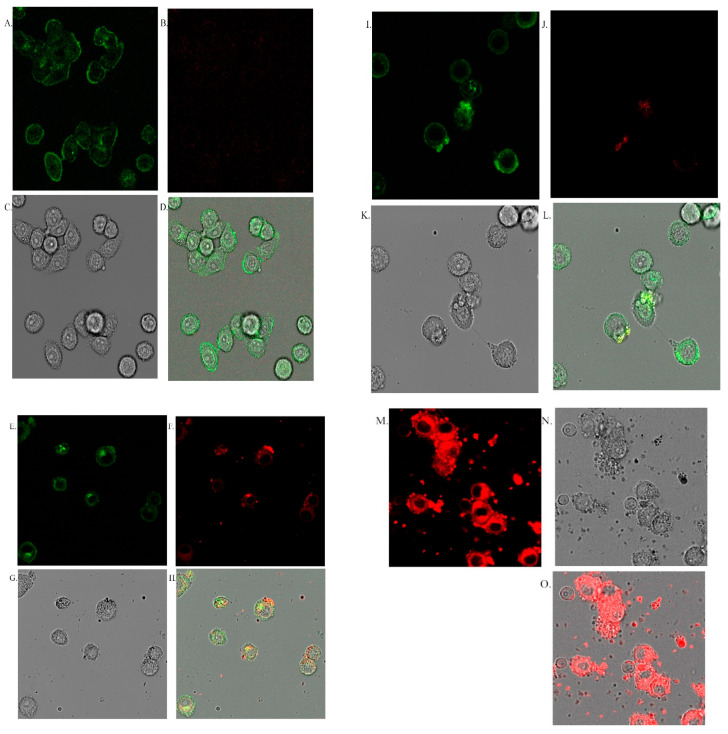
MALM and FC20C induce distinct morphological changes in MDA-MB-468 cells. Cells were treated with MALM or purified 7-hydroxymaltol-3-*O*-*β*-D-glucoside (FC20C) and stained with LysoTracker to visualize lysosomal compartments (**A**,**E**,**I**) and DRAQ7 to assess nuclear morphology and membrane integrity (**B**,**F**,**J**). Corresponding phase-contrast and merged images are shown (**C**,**D**,**G**,**H**,**K**,**L**). Panels (**M**–**O**) show MALM-treated cells at higher intensity. Images were acquired by confocal microscopy at 20× magnification with 3× optical zoom.

To further characterize treatment-associated morphological changes, nuclear integrity and membrane permeability were assessed using DRAQ7 staining and confocal microscopy (Figure 6). Control cells displayed intact nuclear morphology with minimal DRAQ7 uptake (Figure 6A–D). In contrast, MALM- and FC20C-treated cells exhibited pronounced alterations, including redistribution of chromatin toward the nuclear periphery, nuclear condensation, and fragmented nuclear structures (Figure 6E–L). These features were particularly evident at higher MALM concentration (Figure 6M–O), consistent with advanced cellular stress and loss of membrane integrity.

The combined lysosomal and nuclear alterations observed in treated cells are indicative of stress-associated cell death pathways. The nucleolus is highly sensitive to cellular stress and responds by undergoing structural reorganization and fragmentation, events that have been reported following treatment with several anticancer agents, including RNA polymerase I inhibitors, cisplatin, doxorubicin, and mitoxantrone [63,64]. In the present study, these morphological changes occurred alongside caspase-7 and caspase-9 activation and PARP-1 cleavage, supporting a coordinated apoptotic response. Collectively, the imaging and biochemical data suggest that MALM and FC20C compromise lysosomal and nuclear integrity, thereby contributing to apoptotic cell death in TNBC cells.

### 3.5. Role of the PI3K/AKT/mTOR Pathway in MALM- and FC20C-Induced Cytotoxicity

**Figure 7:**
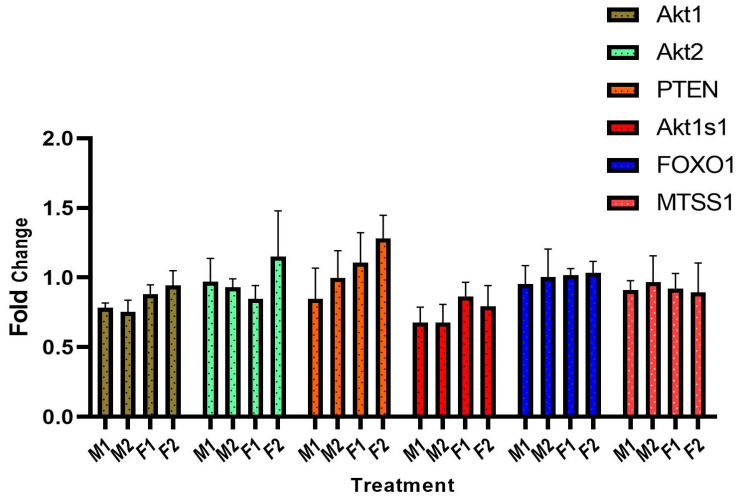
Effects of MALM and FC20C on PI3K/AKT/mTOR pathway-related gene expression in MDA-MB-468 cells. Cells were treated with MALM or purified 7-hydroxymaltol-3-*O*-*β*-D-glucoside (FC20C), and transcript levels of AKT1, AKT2, PTEN, AKT1S1, FOXO1, and MTSS1 were determined by quantitative real-time PCR. Expression levels are shown as fold change relative to DMSO-treated controls. No statistically significant changes in gene expression were observed following treatment.

The PI3K/AKT/mTOR signaling pathway plays a central role in regulating cell survival and proliferation in triple-negative breast cancer [65]. To assess whether MALM or purified 7-hydroxymaltol-3-*O*-β-D-glucoside (FC20C) modulates this pathway at the transcriptional level, the expression of selected pathway-associated genes was evaluated by quantitative real-time PCR (Figure 7). Treatment with MALM or FC20C resulted in only modest changes in the expression of AKT1, AKT2, PTEN, AKT1S1, FOXO1, and MTSS1, none of which exceeded a two-fold difference relative to DMSO-treated controls. These results indicated that MALM- and FC20C-induced cytotoxicity is not primarily mediated through transcriptional regulation of the PI3K/AKT/mTOR pathway. Instead, the absence of significant gene expression changes suggests that alternative mechanisms, such as post-translational signaling events or activation of apoptotic and lysosomal stress pathways, may play a more prominent role in mediating cell death in MDA-MB-468 cells.

### 3.6. Protein–Ligand Interaction

#### 3.6.1. AKT1 Kinase Domain ATP-Binding Pocket

**Figure 8:**
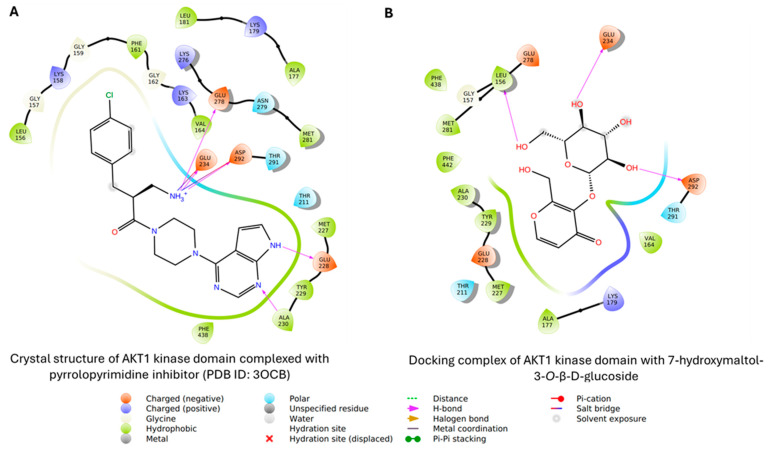
Two-dimensional interaction maps of the AKT1 kinase domain. (**A**) Crystal structure of AKT1 complexed with a pyrrolopyrimidine inhibitor (PDB ID: 3OCB), showing key interactions within the ATP-binding pocket, including hydrogen bonds and electrostatic contacts with hinge-region residues. (**B**) Docking pose of 7-hydroxymaltol-3-*O*-β-D-glucoside within the AKT1 active site, illustrating multiple hydrogen bonding and hydrophobic interactions.

The two-dimensional interaction maps highlight ligand recognition within the ATP-binding pocket of the AKT1 kinase domain. In the co-crystal structure (PDB ID: 3OCB), the pyrrolopyrimidine inhibitor adopts a canonical ATP-competitive pose stabilized by hydrogen bonding with hinge-region residues (Glu228, Glu234, Glu278, Asp292, and Ala230). This anchoring interaction is complemented by extensive hydrophobic contacts involving residues such as Phe438, Tyr229, and Val164, ensuring optimal pocket occupancy (Figure 8A). The protonated amine further contributes to electrostatic stabilization, consistent with high-affinity kinase inhibitors.

Similarly, 7-hydroxymaltol-3-*O*-β-D-glucoside exhibits a hydrogen bond-dominated binding mode, with common residues, Glu234, Asp292, and the backbone of Leu156 via the hydroxyl groups of the glucosyl moiety. Additional contacts with hydrophobic residues (Val164, Met227, Tyr229, Phe438) are observed. The polar sugar moiety remains partially solvent-exposed, suggesting reduced hydrophobic packing but enhanced interaction versatility. The presence of hydrophobic interactions with surrounding residues, such as Leu156, Val164, and Met281 (Figure 8B), further anchors the ligand, reflecting a classical kinase-inhibition pattern observed for ATP-competitive inhibitors [38]. Other polar residues, such as Glu228 and Glu278 (involved in hydrogen bonds in the crystal complex), appear to engage in van der Waals interactions along with residues like Lys179, Thr211, and Thr291, thereby strengthening the binding. Taken together, this binding yields a docking (binding energy) score of −7.40 kcal/mol.

#### 3.6.2. PARP-1

**Figure 9:**
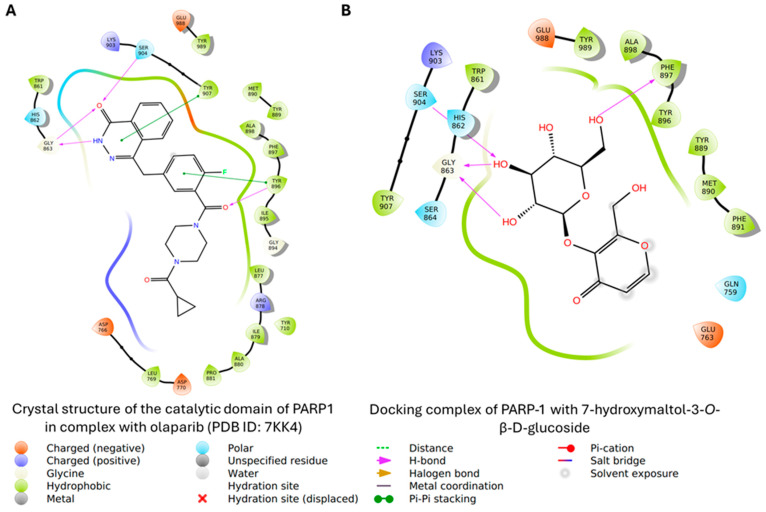
Two-dimensional interaction profiles of the PARP-1 catalytic domain. (**A**) Crystal structure of PARP1 in complex with the reference inhibitor olaparib (PDB ID: 7KK4), showing key hydrogen bonding and hydrophobic interactions within the catalytic pocket. (**B**) Docking pose of 7-hydroxymaltol-3-*O*-β-D-glucoside within the PARP-1 active site, showing extensive hydrogen bonding and polar interactions mediated by its glycosidic moieties.

The interaction maps provide a clear, complementary view of ligand binding within the active site of the PARP-1 catalytic domain. In the co-crystal structure (PDB ID: 7KK4), olaparib adopts a well-optimized binding mode, anchored by key hydrogen bonds to Gly863, Tyr896, and Ser904 that position the ligand effectively within the NAD^+^ pocket. This is further supported by multiple hydrophobic interactions with residues such as Tyr907, Phe897, and Ala898, along with π–π stacking with Tyr896, resulting in a compact and stable ligand–protein complex (Figure 9A).

Interestingly, 7-hydroxymaltol-3-*O*-β-D-glucoside also demonstrates a favorable interaction profile. The ligand forms 2 hydrogen bonds with Gly863 and 1 hydrogen bond with Ser904 through the hydroxyl group of the glycosyl moiety. Both residues are essential for the activity of the enzyme, and their blockade contributes to the inhibition of its catalytic activity. Although its hydrophobic contacts with residues such as Tyr896, Ala898, and Phe891 are less extensive compared to those in the crystal complex, they still contribute to stabilizing the complex (Binding energy -9.07 kcal/mol) (Figure 9B). In particular, the partially solvent-exposed glycosidic moiety appears to enhance interaction flexibility and adaptability within the binding pocket. This interaction pattern reflects a classical PARP1 inhibition mechanism, where precise positioning in the catalytic site ensures high affinity and specificity [66]. This complementary behavior highlights the potential of 7-hydroxymaltol-3-*O*-β-D-glucoside scaffolds as promising starting points for further optimization, particularly in the design of selective and structurally diverse PARP-1 inhibitors.

#### 3.6.3. Caspase 7

**Figure 10:**
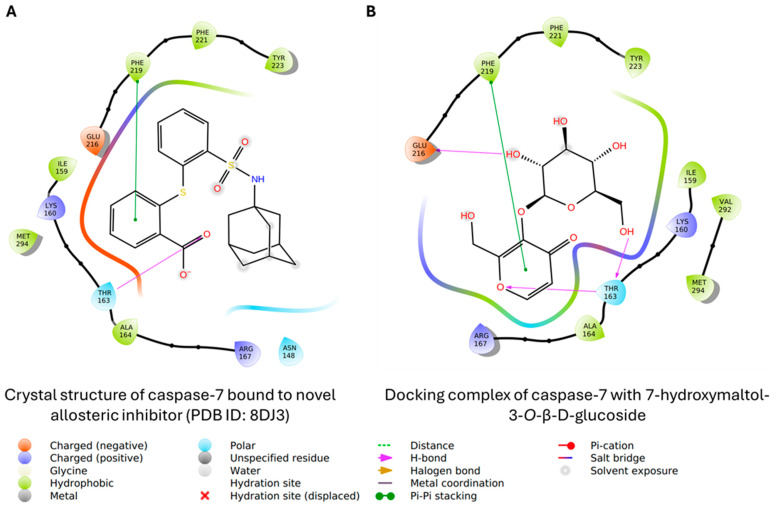
(**A**). Crystal structure of caspase-7 bound to novel allosteric inhibitor (PDB ID: 8DJ3). (**B**). Docking complex of caspase-7 with 7-hydroxymaltol-3-*O*-β-D-glucoside.

The interaction maps provide insight into ligand recognition within the caspase-7 allosteric pocket. In the co-crystal structure (PDB ID: 8DJ3), the reference inhibitor adopts a well-defined conformation stabilized by a combination of hydrophobic and polar interactions (Figure 10A). The aromatic scaffold is engaged in π–π stacking with Phe219, which helps anchor the ligand within the pocket. In addition, hydrogen bonding with residues such as Thr163 and polar contacts with Glu216 contribute to proper ligand orientation. Surrounding hydrophobic residues, including Ile159, Ala164, and Met294, further enhance binding through van der Waals interactions, resulting in a compact and stable complex typical of allosteric caspase inhibitors.

In comparison, 7-hydroxymaltol-3-*O-*β-D-glucoside demonstrates a more flexible and hydrogen bond-driven binding mode within the same site. The ligand forms multiple hydrogen bonds with key residues such as Glu216 via the glycosyl moiety, and Thr163 via both glycosyl and maltol moieties. Similarly, Phe219 is engaged via π-π stacking interaction, thereby supporting the ligand positioning. While hydrophobic interactions with residues like Ile159, Ala164, and Val292 are also present, they are less dominant than in the co-crystallized inhibitor (Figure 10B). The glycosidic moiety appears partially solvent-exposed, which may limit tight hydrophobic packing but supports greater conformational adaptability within the binding pocket [67]. This is reflected in the predicted binding energy score of −6.56 kcal/mol.

### 3.7. ADMET Profile

The analysis revealed that 7-hydroxymaltol-3-*O*-β-D-glucoside possesses a relatively high molecular weight but a reasonable number of hydrogen bond donors (5) and acceptors (9), primarily due to its glycosidic moiety. This structural feature significantly increases its polarity, as reflected by a slightly high topological polar surface area (TPSA: 153.75 Å^2^) (Table 2). While these characteristics enhance aqueous solubility, they negatively impact membrane permeability. Consistent with these properties, the compound was predicted to exhibit low gastrointestinal absorption, suggesting limited oral bioavailability. Furthermore, it was not expected to permeate the blood–brain barrier, which may be advantageous in minimizing central nervous system-related side effects for peripheral targets. The compound was also predicted to act as a substrate of P-glycoprotein, indicating a potential for active efflux that could further reduce intracellular accumulation [68].

In terms of metabolism, 7-hydroxymaltol-3-*O*-β-D-glucoside showed a low likelihood of inhibiting major cytochrome P450 isoforms, suggesting a reduced risk of drug–drug interactions. This is a favorable property in early drug development. However, its high polarity and structural complexity may limit passive diffusion and necessitate transporter-mediated uptake [69].

Drug-likeness evaluation indicated multiple violations of Lipinski’s Rule of Five, mainly due to excessive hydrogen bonding capacity and molecular size. Similarly, Veber criteria were not fully satisfied, primarily because of the high TPSA, reinforcing the prediction of poor oral bioavailability [70]. Despite these limitations, its favorable safety profile and low CYP inhibition potential support its continued investigation, particularly for non-oral or localized therapeutic applications.

## 4. Conclusions

In conclusion, MALM and the isolated pyrone glycoside 7-hydroxymaltol-3-*O*-β-D-glucoside exhibited cytotoxic activity against TNBC cells with limited effects on non-tumorigenic breast epithelial cells. Mechanistic investigations revealed activation of the intrinsic apoptotic pathway, as evidenced by caspase-9 and caspase-7 cleavage and PARP-1 processing, together with pronounced lysosomal and nuclear alterations. While the precise upstream molecular events remain to be defined, the combined biochemical and imaging data indicated that *M. angolensis*-derived constituents induced stress-associated apoptotic cell death. In silico analyses showed that FC20C interacts favorably with AKT1, PARP-1, and caspase-7, suggesting a possible multi-target mechanism of action. Although the compound is predicted to have limited oral bioavailability, it demonstrated a generally favorable safety profile. Overall, these results identify FC20C as a bioactive natural product and provide the first compound-level evidence supporting the anticancer potential of *Maerua angolensis*, highlighting its promise as a lead scaffold for further optimization against aggressive breast cancer subtypes.

## Figures and Tables

**Table 1:** NMR Spectroscopic Data for FC20C (400 MHz, CD_3_OD).

Position	δ_C_, Type	δ_H_ (*J* in Hz)	^1^H−^1^H-COSY	HMBC
2	162.7, C	-	-	-
3	141.3, C	-	-	-
4	176.2, C	-	-	-
5	116.3, CH	6.50, d (5.6)	H-6	3, 4, 6
6	156.2, CH	8.10, d (5.6)	H-5	2, 4, 5
7	56.3, CH_2_	4.75, d (13.6)	-	2, 3
		4.61, d (13.6)		
1′	103.2, CH	4.79, d (7.2)	H-2′	3
2′	76.9, CH	3.28, m	H-1′	1′
3′	73.8, CH	3.38, m	-	4′, 5′
4′	69.9, CH	3.29, m	-	5′
5′	76.4, CH	3.41, m	-	4′
6′	61.2, CH_2_	3.86, dd (11.8, 2.0)	H-5′	4′, 5′
		3.68, dd (11.8, 5.2)	H-5′	

**Table 2:** ADMET and drug-likeness profile of 7-Hydroxymaltol-3-*O*-β-D-glucoside predicted using SwissADME.

Category	Parameter	Prediction/Value
Physicochemical	Molecular weight	High (316.35 g/mol)
	Hydrogen bond donors (HBD)	5
	Hydrogen bond acceptors (HBA)	9
	Topological polar surface area (TPSA)	High (153.75 Å^2^)
	Rotatable bonds	3
	Lipophilicity (LogP)	Low (−2.11)
	Water solubility	High (1.57 × 10^2^ mg/mL)
Absorption	GI absorption	Low
	P-glycoprotein substrate	Yes
Distribution	BBB permeability	No
Metabolism	CYP inhibition (major isoforms)	No significant inhibition predicted
Excretion	Bioavailability score	Low
Drug-likeness	Lipinski’s Rule of Five	Violations (0)
Medicinal Chemistry	PAINS alerts	None

## Data Availability

The data presented in this study are available within the article and its Supplementary Materials. Additional datasets generated and/or analyzed during the current study are available from the corresponding authors upon reasonable request.

## Supplementary Materials

The following supporting information can be downloaded at https://www.mdpi.com/article/10.3390/biom16060861/s1, Figures S1 and S2: Western blot result with band densitometry; Figure S3: Proton NMR spectrum of Fc20c in CD_3_OD; Figure S4: ^13^C-NMR spectrum of Fc20C in CD_3_OD; Figure S5: ^1^ H- ^1^H—COSY Spectrum of Fc20c in CD_3_OD; Figure S6: HSQC spectrum of Fc20C in CD_3_OD; Figure S7: HMBC Spectral of Fc20C in CD_3_OD; Table S1: Bioassay Data.

## Abbreviations

The following abbreviations are used in this manuscript:

**Abbreviation**	**Full Meaning**
AKT	Protein kinase B
DMSO	Dimethyl sulfoxide
HPLC	High-performance liquid chromatography
LC–MS	Liquid chromatography–mass spectrometry
MALM	Methanolic leaf extract of *Maerua angolensis*
PARP-1	Poly(ADP-ribose) polymerase-1
qRT-PCR	Quantitative real-time polymerase chain reaction
TLC	Thin-layer chromatography
TNBC	Triple-negative breast cancer
