# Supplementary material for: A Pyrone Glucoside from Maerua angolensis Induces Caspase-Dependent Apoptosis and Targets AKT1, PARP-1, and Caspase-7 in Triple-Negative Breast Cancer
Source: Biomolecules. 2026 Jun 11;16(6):861. doi: 10.3390/biom16060861 (PMC13297593; doi:10.3390/biom16060861)
Supplement: Supplementary file 1 [file biomolecules-16-00861-s001.zip › biomolecules-4323058-supplementary.pdf]

1. **Western Blot Images**

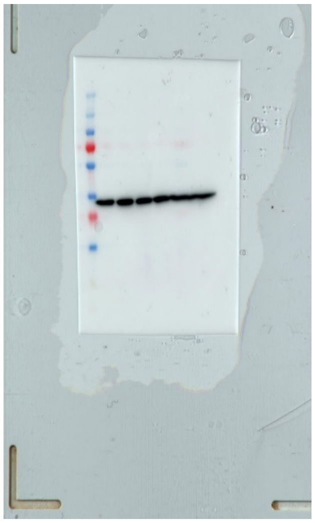

A. Caspase 3 (35KDa)

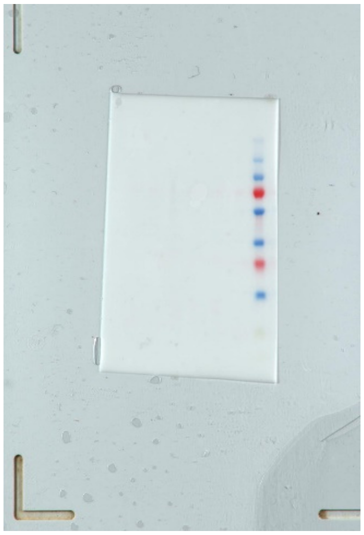

B. Cleaved caspase 3 (17KDa)

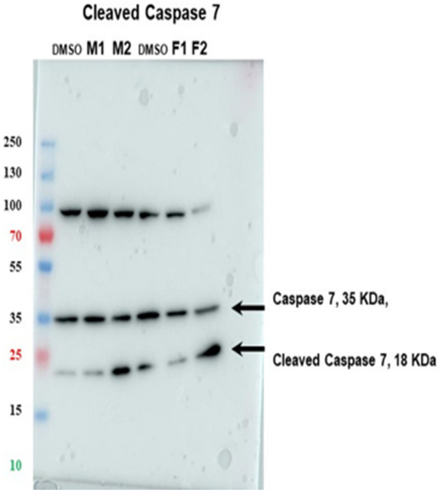

C. Caspase 7 (35 KDa)
D. Cleaved caspase 7 (18KDa)

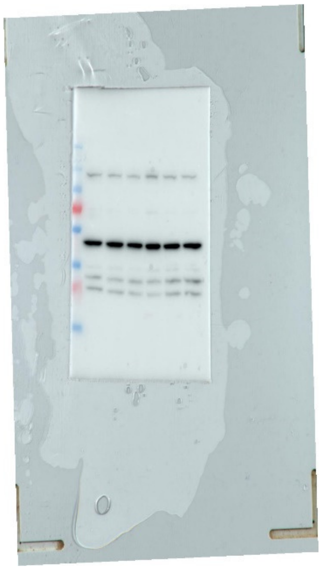

E. Caspase 9 (47KDa)

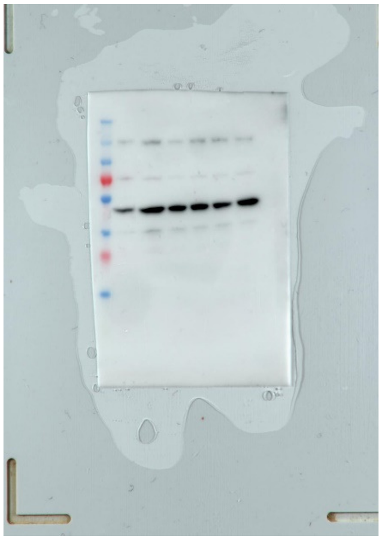

F. Cleaved caspase 9 (37KDa)

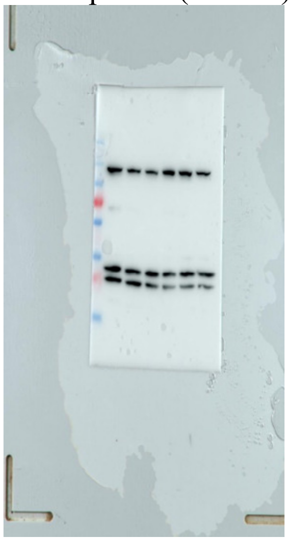

G. PARP (116KDa)

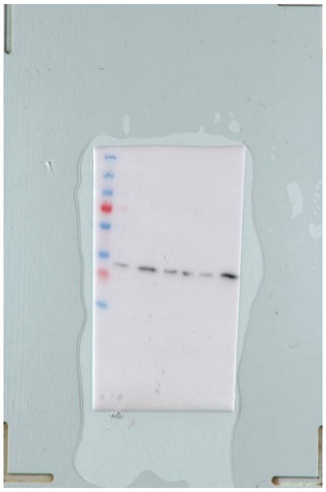

H. Cleaved PARP (24KDa)

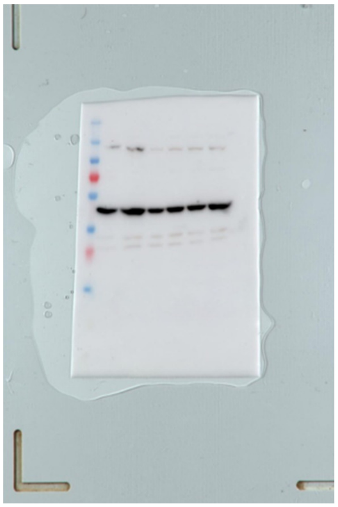

I. Beta-actin (42KDa)

Figure S1: Original Western Blots Images

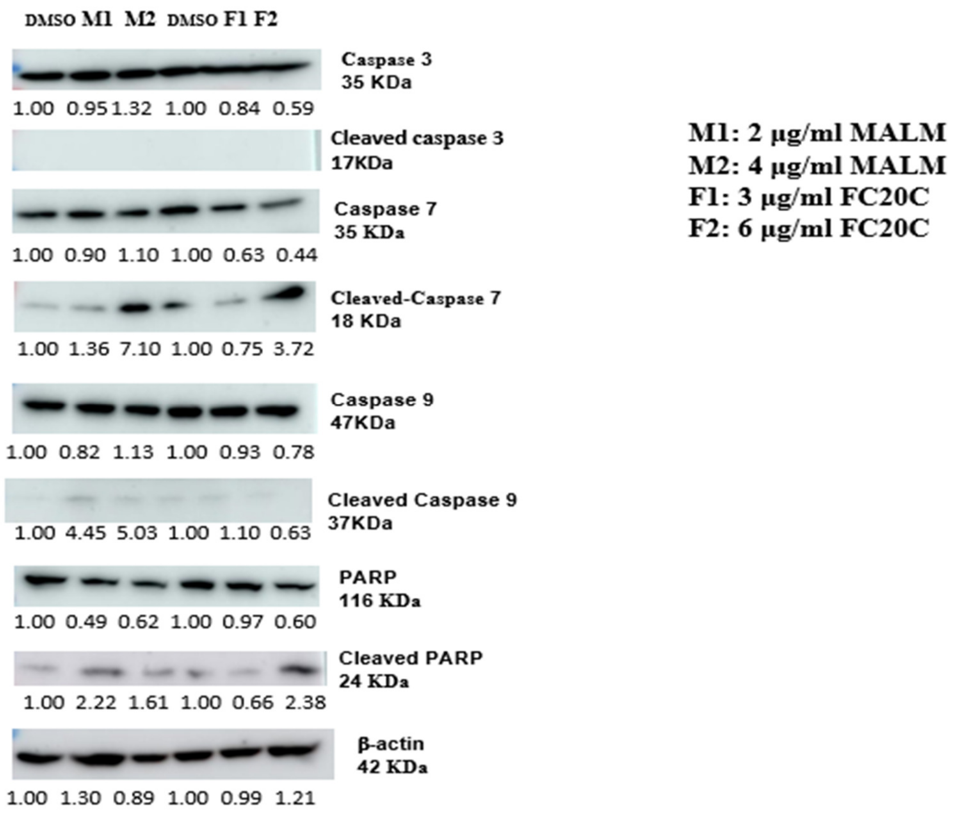

Figure S2: Western blot result with band densitometry.

2. NMR Spectral analysis of the isolated MALM compounds

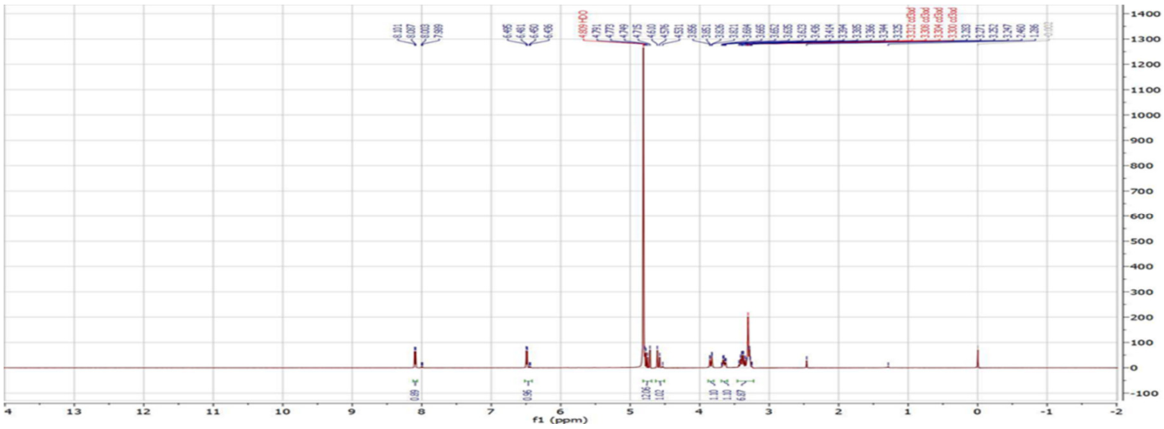

Figure S3: Proton NMR spectrum of Fc20c in CD_3OD

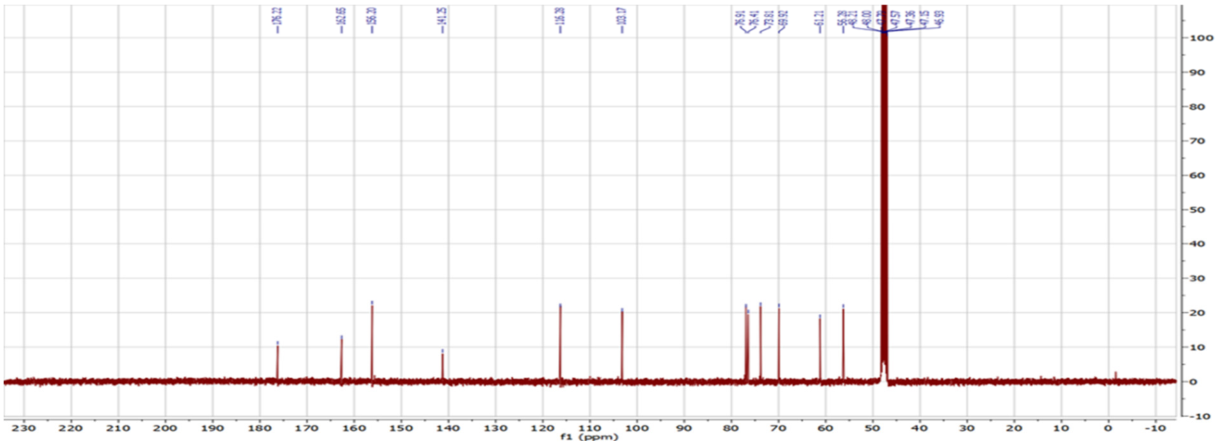

Figure S4: ^{13}C-NMR spectrum of Fc20C in CD_3OD

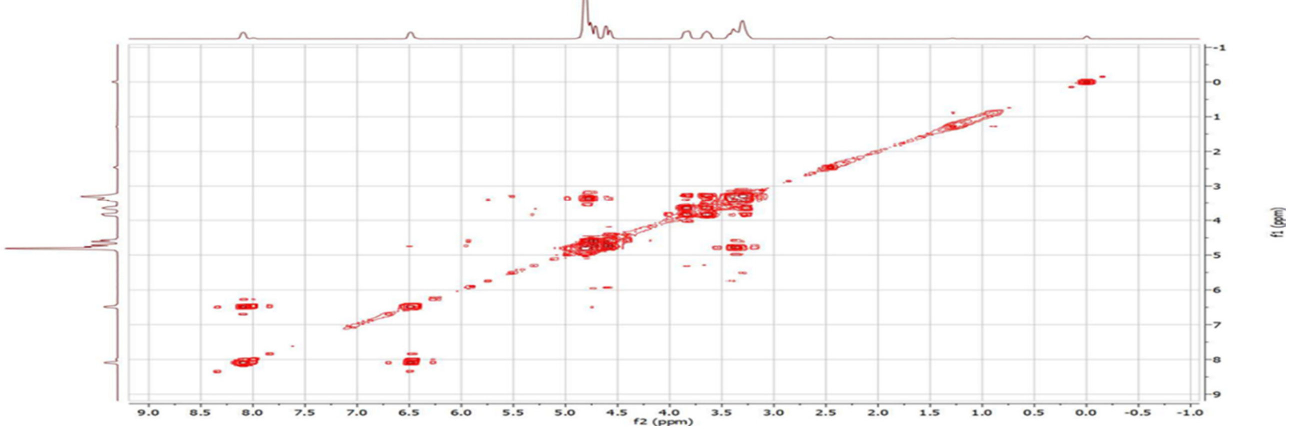

Figure S5: ^{1}H- ^{1}H – COSY Spectrum of Fc20c in CD_3OD

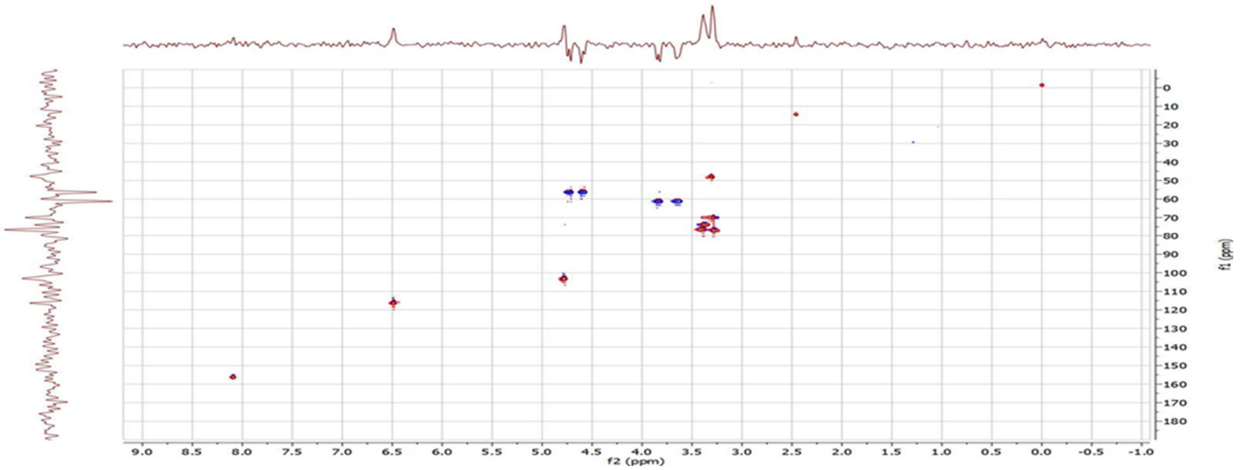

Figure S6: HSQC spectrum of Fc20C in CD_3OD

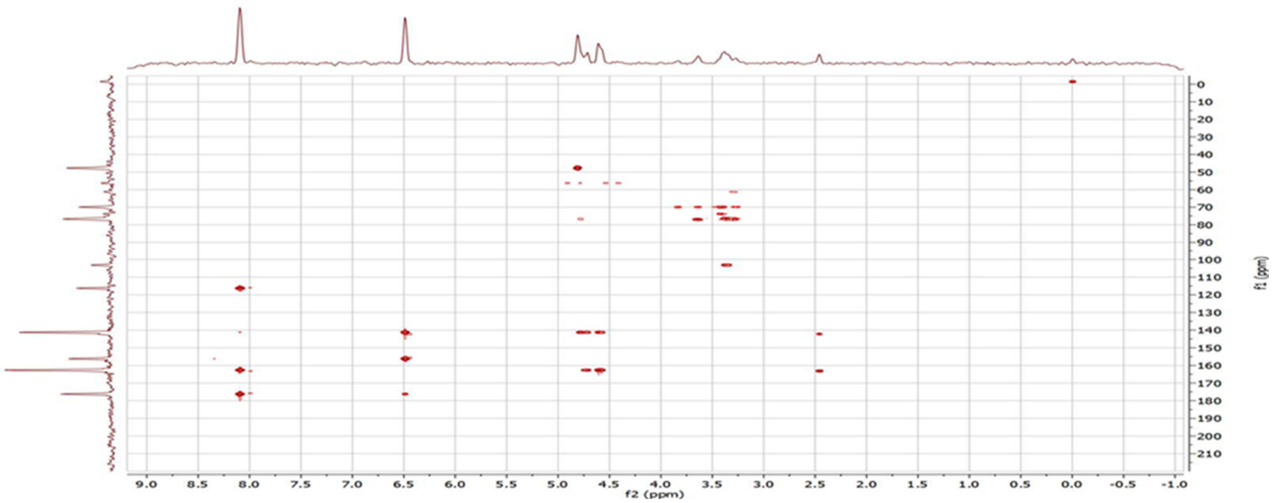

Figure S7: HMBC Spectral of Fc20C in CD_3OD

3. Bioassay (Cytotoxicity)

Table S1: Bioassay Data

**MALM treated MDA-MB 468 Cells**		**FC20C treated MDA- MB 468 Cells**	
Dose (µg/mL)	% Survival	Dose (µg/mL)	% Survival
0.00	100.00	0.00	100.0
Paclitaxel (5ng/mL)	43.82	Paclitaxel (5ng/mL)	27.7
0.781	85.57	0.781	59.1
1.5625	65.94	1.5625	42.9
3.125	54.37	3.125	34.9

6.25	45.44	6.25	32.2
12.5	37.94	12.5	26.7
25	32.29	25	18.3
50	23.21	50	17.8
100	16.69	100	15.6

**MALM treated MCF10A Cells**		**FC20C treated MCF10A Cells**	
Dose (µg/mL)	% Survival	Dose(µg/mL)	% Survival
0	100	0.00	100.00
0.781	86	0.78	92.91
1.5625	91	1.56	75.30
3.125	100	3.13	66.78
6.25	99	6.25	62.35
12.5	71	12.50	58.47
25	65	25.00	65.34
50	58	50.00	47.62
100	26	100.00	41.20
